# Path Analysis Reveals the Direct Effect of PCB28 Exposure on Cognitive Dysfunction in Older Chinese Females

**DOI:** 10.3390/ijerph19126958

**Published:** 2022-06-07

**Authors:** Chenwei Pan, Huijuan Zhao, Qiaoling Du, Yong Xu, Dajun Tian, Shuo Xiao, Haiyin Wang, Xiao Wei, Chunfeng Wu, Yuanyuan Ruan, Chunhua Zhao, Gonghua Tao, Weiwei Zheng

**Affiliations:** 1 School of Public Health, Medical College of Soochow University, Suzhou 215123, China; chwpan@suda.edu.cn (C.P.); childhealth@suda.edu.cn (Y.X.); 2 Key Laboratory of Public Health Safety, Ministry of Education, Department of Environmental Health, School of Public Health, Fudan University, No. 130 Dongan Road, Shanghai 200032, China; 18211020123@fudan.edu.cn; 3 Key Laboratory of Health Technology Assessment, National Health Commission of the People’s Republic of China, Fudan University, Shanghai 200032, China; 4 Department of Obstetrics, Shanghai First Maternity and Infant Hospital, Tongji University School of Medicine, Shanghai 200040, China; qldu2004@126.com; 5 Department of Epidemiology and Biostatistics, College for Public Health and Social Justice, Saint Louis University, 3545 Lafayette Ave, St. Louis, MO 63104, USA; dajun.tian@slu.edu; 6 Department of Pharmacology and Toxicology, Ernest Mario School of Pharmacy, Environmental and Occupational Health Sciences Institutes, Rutgers University, Piscataway, NJ 08854, USA; sx106@pharmacy.rutgers.edu; 7 Department of Health Technology Assessment, Shanghai Health Development Research Center, Shanghai 200032, China; why0522@126.com; 8 Department of Occupational and Environmental Health, School of Public Health, Guangxi Medical University, Nanning 530021, China; weixiao@gxmu.edu.cn; 9 Shanghai Municipal Center for Disease Control & Prevention, No. 1380 West Zhongshan Road, Shanghai 200336, China; wuchunfeng@scdc.sh.cn; 10 NHC Key Laboratory of Glycoconjugates Research, School of Basic Medical Sciences, Fudan University, Shanghai 200032, China; yuanyuanruan@fudan.edu.cn; 11 Department of Biochemistry and Molecular Biology, School of Basic Medical Sciences, Fudan University, Shanghai 200032, China; 12 Education and Training Department, the Affiliated Suzhou Hospital of Nanjing Medical University, Suzhou 215001, China; 13 Technology Department, The Affiliated Suzhou Hospital of Nanjing Medical University, Suzhou Municipal Hospital, Gusu School, Nanjing Medical University, 26 Daoqian Street, Suzhou 215001, China

**Keywords:** path analysis, polychlorinated biphenyls, cognitive dysfunction, older females, Abbreviated Mental Test

## Abstract

Background: Research indicates that exposure to polychlorinated biphenyls (PCBs) can cause neurobehavioral impairments in neonates and adults, but the way specific PCBs’ congeners impact cognition functions at a low exposure level in a real-life co-exposure system remains poorly understood. This study aimed to investigate the association of PCBs burden with cognition function among elderly adults. Methods: Based on the Weitang Geriatric Diseases study (2014–2015), the current study measured the plasma concentrations of six indicator-PCBs by GC-MS/MS and assessed the cognitive dysfunction (CoD) via an Abbreviated Mental Test in 266 participants (ages 61–90). Sequential logistic regression was used to analyze the effects of PCBs on cognition functions. Female participants aged less than or equal to 80 years were selected, and path analysis was used to determine the direct or indirect impacts of co-exposure PCBs on CoD by structural equation modeling. Results: After sequential adjustments to potential confounding factors and correction by the Bonferroni, no statistically significant correlation between PCBs exposure and CoD was found in participants (*p* > 0.05). However, in the co-exposure system, after controlling for co-exposures and confounders, exposure to PCB28 had a direct effect on CoD in females aged between 61 and 80, with a factor load of 0.670. Conclusions: After adjusting for the co-exposures and confounders, exposure to PCB28 can directly increase the risk of cognitive impairment in older Chinese females.

## 1. Introduction

Cognitive impairment has been well identified as a critical health risk factor for older adults. Mild cognitive impairment, which is the intermediate stage between the normal aging cognitive changes and dementia, is currently considered as a key risk factor to dementia [1]. Dementia affects nearly 35.6 million people worldwide, and this number will increase to 115.4 million by 2050 [2]. In China, the prevalence of dementia, cognitive dysfunction (CoD) without dementia, and mild cognitive impairment in the older population is 2.8%, 12.7%, and 14.71%, respectively [3,4,5]. There is an urgent need to identify the risk factors for mild cognitive impairment in older adults.

Environment pollutants have been shown to impair cognition functions, either alone or in combination with genetics and aging [6]. As one of the 12 POPs initially identified by the Stockholm Convention [7], polychlorinated biphenyls (PCBs) used to be intensively produced and widely used globally as heat exchange fluids and insulating oils [8]. Although China has banned the use of PCBs in manufactured power capacitors and restricted the import of PCB power devices since 1974 [9], the production of PCBs has increased rapidly since 2000 from the by-products of industrial processes such as E-wastes (51%) and volatilized pigment/paintings (10%) [10,11].Thus, PCBs are detectable in the atmosphere (from <10 to >3000 pg/m^3^), soil (from <1 to >1000 ng/g), river water (from <5 to >500 ng/g) [12], and food (from 46.7 to 628.7 pg/g) [13], with the primary exposure route of PCBs in humans being contaminated foods [14].

PCBs are a group of organochlorine compounds synthesized by biphenyl chlorination, with a total of 209 homologs [15]. According to the differential physical–chemical characteristics, the 209 homologs of PCBs can be categorized into 12 dioxin-like PCBs (dl-PCBs) and 197 non-dioxin-like PCBs (ndl-PCBs) [15]. They can also be divided into the lower-chlorination PCBs (LPCBs, with 5 chlorine atoms or less) and higher-chlorination congeners (HPCBs) based on the number of chlorine atoms [16]. Since it is impractical to test all 197 ndl-PCBs, Beck et al. (1985) suggested that research studies can primarily focus on 6 indicator PCB congeners, including PCB28, 52, 101, 138, 153, and 180 [15].

The neurotoxicities of PCBs have been a public health concern for decades [17]. In both epidemiological and animal studies, it has been shown that intrauterine exposure to PCBs can cause neurobehavioral impairments in neonates [18,19]. In an American study on older residents, the increased serum concentrations of total PCBs were positively associated with a decrease in verbal learning [20]. Another study reported the dose-dependent neurocognitive deficits in certain aspects of memory and learning ability in women with a previous exposure history of PCBs [21]. Subsequently, some researchers analyzed in detail the PCB burden of PCB-exposed workers and revealed that LPCBs had a negative effect on word fluency, and HPCBs and dl-PCBs had a negative impact on aiming and line tracking [22]. However, it is poorly defined that which specific PCBs congeners affect cognition functions at a long-term low exposure level.

The mechanisms of altered dopamine (DA) signaling, disruption of thyroid hormone signaling, perturbation of intracellular Ca^2+^ dynamics, and oxidative stress have also been reported as possible mechanisms of PCB-induced neurotoxicity [17]. As a LPCB, PCB28 has low vapor pressure, poor aqueous solubility, and high resistance to biological degradation [23]. In adult rats, exposure to PCB28 resulted in long-lasting deficits in learning, and this effect may be female specific [24]. It was also found that lower-chlorination non-dioxin-like PCBs can act as a GABA_A_ receptor agonist to disrupt brain development, motor coordination, learning, and memory [25], providing a possible molecular mechanism to link PCB28 exposure and cognitive dysfunction.

In the present study, we selected community-dwelling older adults as research subjects in order to investigate the association of PCB burden with cognition functions among 266 adults aged 60 years or older. The plasma concentrations of six indicator-PCBs were measured to determine the PCB burden. In addition, a questionnaire survey was performed to test subjects’ cognition function. We used the logistic regression method to analyze whether exposure to PCBs was a risk factor of cognitive impairment, and then structural equation modeling (SEM) was used for path analyses, which allowed us to determine the co-exposure effects of PCBs and whether these effects were direct or indirect. This also helped us to identify the contribution of individual PCBs within the co-exposure of all indicator-PCBs. This study provides epidemiological evidence to show the toxic effects of PCBs on cognitive functions in elderly people; moreover, the final path analyses determined the impacts of co-exposure to PCB28, 101, 138, 180 and revealed that PCB28 directly increases the risk of cognitive impairment in elderly females.

## 2. Materials and Methods

### 2.1. Study Population

This study was conducted based on the Weitang Geriatric Diseases study, a cross-sectional, community-based study that aimed to investigate the patterns, predictors, and burdens of health among elderly residents aged 60 years or older in the East China region [26]. Weitang Town, located in the district of Xiangcheng in the city of Suzhou, is an old industrial town on the southeastern coast of China. This town covers a total area of 39.36 km^2^. The six major industries in Weitang are hardware and plastic molds, auto parts, machinery, health care products, mechatronics, and purification equipment.

The Weitang Geriatric Diseases study recruited 6030 elder adults over 60 years of age from August 2014 to February 2015. Participants were excluded if they were: (1) younger than 60 years of age; (2) moved from Weitang Town to another place; (3) were living in Weitang Town for less than 6 months; or (4) died during the study. In summary, 5613 subjects were included in the Weitang Geriatric Diseases study database, with 4579 of them having completed a questionnaire containing the Abbreviated Mental Test and having provided blood samples. A pilot study was conducted to explore the association of the general levels of PCBs in the blood of community-dwelling older adults with their cognition functions. Therefore, a total of 266 consecutive samples from the database were selected to measure the plasma concentrations of 6 indicator-PCBs.

The Weitang Geriatric Diseases study was carried out in accordance with the principles of the Declaration of Helsinki and approved by the Institutional Review Board (IRB) of Soochow University. At the recruitment stage of this study, all participants gave written informed consent.

### 2.2. Cognitive Functions Outcomes

The Abbreviated Mental Test (AMT) (Hong Kong version) [27], was used to assess the CoD in this study. As previously described [26], some culturally specific items were modified to improve the relevance to the local context. The final ten-item scale included age, current time, year, place, features identification, date of birth, the National Day of China, the president of China, counting down from 20, and recall of an address. Correctly answering an item resulted in a score of 1, and the maximum total score was 10. The total score was then grouped based on the criteria of normal cognitive function (>7) or CoD (≤7) [28].

### 2.3. PCBs Concentrations in the Plasma Samples

Blood samples were stored at −80 °C for 3 years until measurement in the Shanghai Municipal Center for Disease Control and Prevention. The preparation of plasma samples was as follows: an aliquot of 0.2 mL plasma was mixed with 3 mL ethyl acetate/n-hexane (*v*/*v*, 1:1) and centrifuged. Each sample was extracted in twice. The supernatant was transferred to a new tube, then the solvent was removed under a stream of nitrogen and with 40 °C water bath. The dried sample was redissolved in 0.4 mL N-hexane and 0.4 mL sulfuric acid, successively, for purification and then centrifuged. The 0.2 mL supernatant was desiccated with anhydrous sodium sulfate for subsequent analyses.

The identification and quantification of plasma PCBs levels were analyzed using a Thermo Scientific TRACE 1300 Series gas chromatograph coupled with a Thermo Scientific TSQ 8000 EVO Triple Quadrupole mass spectrometer (Thermo Fisher Scientific, San José, CA, USA). The gas chromatograph was fitted with a TG-5MS column (30 m long, 0.25 mm i.d., 0.25 μm film thickness; Thermo Fisher Scientific, San José, CA, USA). The carrier gas was high purity helium at a flow rate of 1.0 mL/min. Samples were injected at 1 µL in splitless mode. The oven temperature was started at 150 °C (held for 1 min), then increased at a rate of 20 °C/min till 200 °C and held for 0 min, then increased at a rate of 8 °C/min to 300 °C and held for 5 min. The mass spectrometer was used in the electron impact source mode, and the collision gas was argon. The ion source temperature was 280 °C, and the transmission line temperature was 300 °C.

We used 200 µL of a mixture of 6 indicator-PCBs as the standard solution to draw the standard curve, with the linear range from 0.05 to 5.0 ng/mL and the correlation coefficient R^2^ > 0.9999. A blank method sample was included with every batch of serum samples. The concentrations of the analytes in the blank samples were not detected; therefore, we did not correct the concentrations in the samples. The recovery rate of 6 indicator-PCBs ranged from 68.40% to 135.00%. The relative standard deviation (RSD) was 4.4% to 10.2%. The limit of detection (LOD) was regard as 60% of the lower limit of quantitation, which was 0.03 ng/mL. The value was assigned as 0 in the plasma sample when PCBs were not detectable. The “total lipid” concentrations were calculated via short formula [29] to adjust the PCB measurements in the plasma. The plasma concentrations of PCBs were shown as lipid-adjusted concentrations. Concentrations below LOD were reported as not detected (ND).

### 2.4. Statistical Analysis

We performed the statistical analyses using R (version 4.0.2, R Foundation for Statistical Computing, Vienna, Austria). In descriptive analyses, continuous variables were expressed by their median (interquartile range, IQR) and compared with the Mann-Whitney U test; categorical variables were expressed as a number (%) and compared by chi-square test. The *p*-value < 0.05 was considered as statistically significant.

Sequential logistic regression analysis: Since our research subjects were a non-occupational exposure population, over 50% of the samples had an exposure level for the 6 indicator-PCBs that was below LOD. Therefore, PCB congeners with less than at least 10% of measures above the LOD were excluded from further analysis. We split up the exposure of PCB 28, 101, 138, 153, LPCBs, HPCBs, and ∑PCBs in dichotomous variable (>LOD vs. <LOD) and included them as dummy variables in the models. Sequential logistic regression models were used to preliminarily explore the association between the exposure of PCBs and CoD.

(1) Model 0 was univariate logistic regression.
(2) Model 1 adjusted for baseline covariates including age and sex.
(3) Model 2 additionally adjusted for education level (formal education vs. without formal education), monthly income (≤1 k, 1.01–3 k, >3 k), marriage (living with a spouse vs. living without a spouse), offspring (yes vs. no), sleep quality (poor vs. general vs. well), and sleep duration.

In order to adjust for multiple comparisons, a Bonferroni-holm test was used to find adjusted significant differences.

Path analysis: Previous studies have shown that the effects of PCBs on cognition function may be sex specific [24]. Therefore, we conducted a subgroup analysis of all female participants. We then excluded participants aged above 80 years from the predefined subgroup, because the magnitude of the relationship between neuropsychological function and age remained stable from ages 65 to 80 but was stronger above the age of 80 [30].

**Figure 1:**
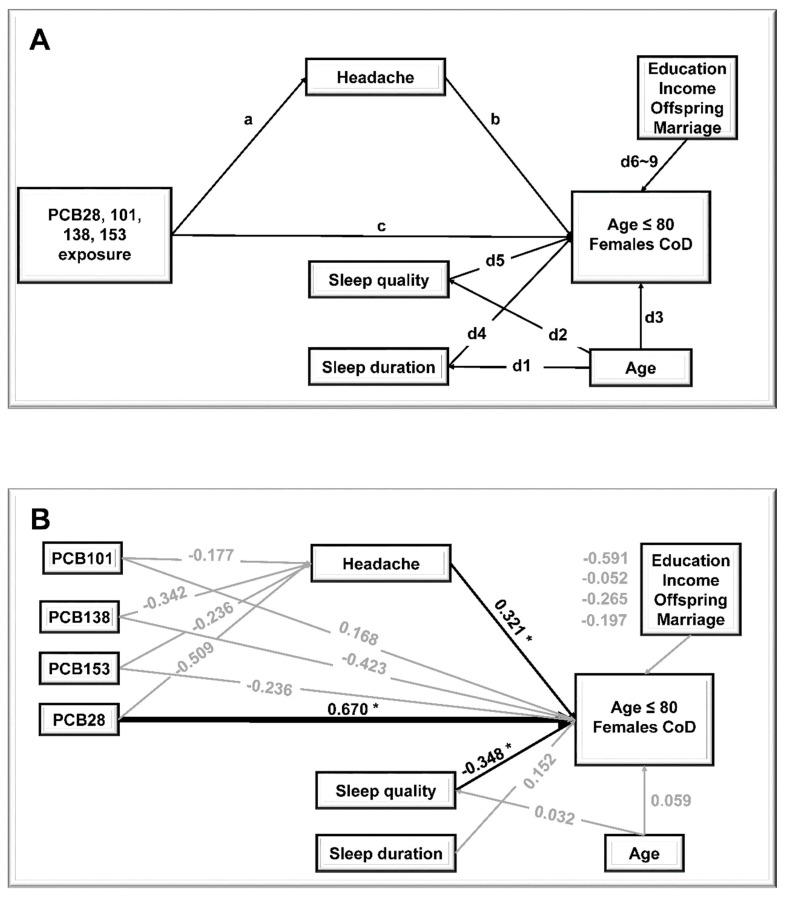
Process of path analysis by structural equation modeling (SEM). (**A**) The hypothesis model of the exposure of 4 PCBs (PCB 28, PCB 101, PCB 138, and PCB 153), female participants’ CoD and confounders; (**B**) the direct effect of exposure to PCB 28 on CoD in female participants with ages below and equal to 80. * and blank line indicate *p* value < 0.05. The gray line indicates *p* value ≥ 0.05. Line thickness represents effect size (the thicker the line, the stronger the effect’s absolute value).

To simulate the exposure of mixtures of environmental toxins, PCB 28, 101, 138, and 153 were included in the research hypothetical system and analyses framework (Figure 1A). SEMs were conducted for path analysis using R package “lavaan”. Models were adjusted for education level, monthly income, marriage, and offspring. The final model was fit by removing PCBs that were not significantly (*p* value ≥ 0.05) associated with CoD. Good model fit was assessed as a chi-square *p* value above 0.05, a root mean square error of approximation (RMSEA) below 0.05, and a comparative fit index (CFI) above 0.95 [31].

The path coefficients were calculated using SEM in R (version 4.0.2) by the ‘lavaan’ package. The a and b estimated the indirect effects of exposure to 4 PCBs on CoD through headaches; c estimated for direct effects between exposure to 4 PCBs andCoD. d1 and d2 estimated for the confounding factors’ effects on Sleep duration and Sleep quality, respectively; d3–9 estimated for the confounding factors’ effects onCoD.

## 3. Results

### 3.1. Basic Characteristics of the Participants

The characteristics, lifestyle, and health conditions of participants in this study are shown in Table 1, and these indexes had no statistical difference compared with the whole population of participants as previously described [32].

A total of 266 elderly adults living in communities were included, with the age of participants ranging from 61 to 90; the median (IQR) age was 67 (IQR 63–74). The 266 recruited participants were placed into either the Normal group (N = 211) or the CoD group (N = 55) based on their AMT scores. The median age of participants in the Normal group was 66 (IQR 63–71), which is significantly younger than the median age of participants in the CoD group at 75 (IQR 65–81, *p* < 0.001). Compared to the Normal group, participants in the CoD group were predominantly female (*p* < 0.001).

### 3.2. Plasma Concentrations of PCBs

The plasma concentrations of six indicator-PCBs are shown in Table 2. PCB101 had the highest detection rate of 41.35%, while PCB52 had the lowest detection rate of 0.38%. The median concentration of the six indicator-PCBs was 12.69 ng/g lipid. The plasma concentrations of HPCBs were generally higher than LPCBs, with PCB180 showing the highest plasma concentration at 18.25 ng/g lipid (IQR 5.68–96.63), followed by PCB138 at 15.45 ng/g lipid (IQR 8.60–30.67) and PCB28 at 8.95 ng/g lipid (IQR 8.27–10.10). PCB52 and PCB180 had fewer than 10% of measures above the LOD, and therefore they were excluded from further analyses.

### 3.3. Association between PCBs Burden and CoD

The results of the association analyses between PCBs and CoD are shown in Table 3. After being adjusted by age, sex, education level, monthly income, marriage, offspring, sleep quality, and sleep duration, there was a significant difference (*p* < 0.05) for the exposure of PCB28 between the Normal group and CoD group. However, after corrected by the Bonferroni, there was no significant difference (*p* > 0.05).

### 3.4. Path Analyses of PCBs Burden and CoD Association

The research hypothetical system and analyses framework are shown in Figure 1A. We included four PCBs (PCB28, PCB101, PCB138, and PCB153) in the basal model, which were the congeners with more than at least 10% of measures above the MDL.

In Figure 1B and Supplementary Table S1, we listed the standardization regression coefficients (factor loads) among variables in the final model. Results indicated that exposure to PCB28 had a direct effect on CoD in females aged 80 and below, with the factor load at 0.670. This effect size indicated that exposure to PCB28 was associated with an increased risk of CoD by 0.670 points after controlling for the other PCBs, age, education level, monthly income, marriage, offspring, headache, sleep quality, and sleep duration. Meanwhile, none of the examined PCBs were indirectly associated with CoD for the variable of mediation of headache.

The model was well fitted in these participants assessed by χ^2^ *p*-value, CFI and RMSEA.

## 4. Discussion

The results of our study indicate that exposure to PCBs may be associated with CoD in elderly people over 60 years of age. More specifically, after adjusting for the co-exposures and other confounders, exposure to PCB28 may directly heighten the risk of cognitive impairment in elderly females aged 80 or younger, which may suggest the real effects of PCBs rather than age-related cognitive decline. More in-depth mechanistic studies are required to further evaluate the influence of PCBs exposure on CoD.

Compared with other regions in China, the exposure level of PCBs in our study population was at an average level [33,34]. Compared with other countries, the exposure level of PCBs in our study population was at a relativly low level [35,36,37,38,39,40,41]. The plasma concentrations of PCBs were related to the dietary intake pattern of local residents [15,42] and decreased with age [43,44]. This low exposure level to PCBs may be caused by different dietary patterns in different countries as well as the different ages of study subjects (Table 4).

So far, the results of previous studies have been inconsistent regarding the effects of PCBs on cognition functions. A report from Canada [45] showed that exposure to PCB153 was associated with reduced mean cognitive performances. A survey based on the National Health and Nutrition Examination Survey (NHANES) observed an opposite trend of cognitive functioning when PCB153 exposure occurred simultaneously with that of PCB74, 118 or 146 (*β* = 0.200, 95% *CI*: 0.05, 0.35; *p* < 0.05) [37]. These epidemiological studies have severals limitations. For example, Medehouenou et al. did not consider the co-exposures [45], and Przybyla et al., did not comprehensively detect six indicator-PCBs [37]. The present study was designed to evaluate such associations among Chinese aged 60 and above after controlling for co-exposure and confounders. In our study, after sequential adjustments of potential confounding factors and correction by Bonferroni, no significant correlation can be proven (*p* > 0.05). After controlling for co-exposures and confounders, we did not find that exposure to PCB153 was directly or indirectly associated with cognitive dysfunctions in this co-exposure system (*β* = 0.140, SE = 0.540; *p* > 0.05). Only exposure to PCB28 was directly associated with cognitive dysfunction (*β* = 0.670, SE = 0.339; *p* < 0.05). Furthermore, ‘Headache’ produced no mediated effect to suggest that exposure to PCBs increased prevalence of CoD.

Evidence has revealed that PCBs, as neurotoxicants, could cause deficits in cognitive flexibility, working memory, and inhibitory control by influencing intracellular signaling and disruption of Ca^2+^ homeostasis and neurotransmitters [46,47]. PCB28 belongs to LPCBs and has a lower potential to bioaccumulate in the body [16]. PCB28 also belongs to ortho-substituted PCBs and the neurotoxic equivalent (NEQ) is 0.298 [48]. Together with the results from previous studies, we found that PCB28 could influence cognitive dysfunctions after controlling for PCB 101, 138, 153, and other confounders. Further studies, however, are necessary investigate the potential molecular mechanisms.

There are some limitations in this study. The self-reported variables in the questionnaire may lead to recall biases. The gender stratified analysis should be further verified in a larger sample size. In addition, the detection rates of the 6 indicator-PCBs were relatively low. For PCB52, the NEQ was higher than that of the other five PCBs [48], but the plasma concentration appeared too low in the participants in our study to effectively observe the effect of PCB52 on cognitive impairment. In the current study, the low detection rates may be caused by the insufficient sample size or the PCBs low exposure to the study population. The lack of internal standardization may also affect the accuracy of the detection results. In addition, all samples were selected from a town in China, and as such the extrapolation of the results was limited. Furthermore, it is true that chromatograms do not always robustly allow the differentiation between surrounding noise and measured value at low levels, which can cause false positives and false negatives. Therefore, studies with a larger and more representative population should be carried out, and internal standardization should be implemented in a follow up and future measurements to further validate the results and conclusions in this study.

## 5. Conclusions

This study, for the first time, reveals that after controlling for the co-exposure to indicator-PCBs and confounders, exposure to PCB28 can directly heighten the risk of cognitive impairment in Chinese elderly females. This identified effect of PCB28 suggests that mechanistic research on neurotoxicity and control strategy should be focused on PCB28. Our study establishes the analysis framework that can be used to determine the effects of a mixture of chemicals and identify the contribution of individual chemicals from exposure to the mixture. These results provide a scientific basis and case for the identification and prevention of environmental pollutants in CoD among the elderly people living in communities so as to reduce the potential risk of CoD.

## Figures and Tables

**Table 1:** Participants characteristics (N = 266).

	Total	Normal (N = 211)	CoD ^a^ (N = 55)	*p* ^b^
Age (years) ^d^	67 [63, 74]	66 [63, 71]	75 [65, 81]	<0.001
Sex (%)				<0.001
Male	123 (46.2)	111 (52.6)	12 (21.8)	
Female	143 (53.8)	100 (47.4)	43 (78.2)	
Education level (%)				<0.001
Without formal education	131 (49.2)	89 (42.2)	42 (76.4)	
Primary school	106 (39.8)	94 (44.5)	12 (21.8)	
Middle school	25 (9.4)	24 (11.4)	1 (1.8)	
High school	4 (1.5)	4 (1.9)	0 (0.0)	
College	0 (0.0)	0 (0.0)	0 (0.0)	
Monthly income CNY (%)				0.004
≤1 k	166 (62.9)	121 (57.9)	45 (81.8)	
1.01–3 k	83 (31.4)	74 (35.4)	9 (16.4)	
>3 k	15 (5.7)	14 (6.7)	1 (1.8)	
Living with spouse (%)				0.058
Without	43 (16.2)	29 (13.7)	14 (25.5)	
With	223 (83.8)	182 (86.3)	41 (74.5)	
Offspring (%)				0.222
No	128 (48.1)	97 (46.0)	31 (56.4)	
Yes	138 (51.9)	114 (54.0)	24 (43.6)	
Smoking status (%)				<0.001
Never smoker	171 (64.3)	124 (58.8)	47 (85.5)	
Current/former smoker	95 (35.7)	87 (41.2)	8 (14.5)	
Habitual alcohol drinker (%)				0.020
No	203 (76.3)	154 (73.0)	49 (89.1)	
Yes	63 (23.7)	57 (27.0)	6 (10.9)	
Sleep quality (%)				0.009
poor	28 (10.5)	16 (7.6)	12 (21.8)	
general	35 (13.2)	28 (13.3)	7 (12.7)	
well	203 (76.3)	167 (79.1)	36 (65.5)	
Sleep duration(h) ^d^	264 ^c^	210 ^c^	54 ^c^	
	9.0 [8.0, 9.0]	8.0 [8.0, 9.0]	9.0 [8.0, 10.0]	<0.001
Headache (%)				0.043
No	208 (78.2)	171 (81.0)	37 (67.3)	
Yes	58 (21.8)	40 (19.0)	18 (32.7)	
Diabetes (%)				0.057
No	241 (90.6)	187 (88.6)	54 (98.2)	
Yes	25 (9.4)	24 (11.4)	1 (1.8)	
Hypertension (%)				0.916
No	241 (90.6)	187 (88.6)	54 (98.2)	
Yes	141 (53.0)	111 (52.6)	30 (54.5)	
AMT score ^d^	9.0 [8.0, 10.0]	10.0 [8.0, 10.0]	6.0 [5.0, 7.0]	<0.001

^a^: CoD = Cognitive dysfunction. ^b^: *p* values were obtained from Mann-Whitney U test or chi-square test. ^c^: two participants were missing in ‘Sleep duration’. ^d^: median [IQR].

**Table 2:** Plasma PCBs levels (ng/g lipid) of the participant sample (N =266).

PCBs	Median [IQR]	Range	>MDL ^a^ (n)	>MDL (%)
PCB28 ^b^	8.95 [8.27, 10.10]	ND-127.07	37	13.91%
PCB52 ^b^	162.74	ND-162.74	1	0.38%
PCB101 ^b^	11.30 [8.50, 16.10]	ND-247.46	110	41.35%
PCB138 ^c^	15.45 [8.60, 30.67]	ND-309.88	25	9.40%
PCB153 ^c^	10.96 [6.95, 15.92]	ND-283.12	22	8.27%
PCB180 ^c^	18.25 [5.68, 96.63]	ND-294.27	4	1.50%
∑PCBs ^d^	12.69 [8.91, 21.89]	ND-1424.54	140	52.63%

^a^: MDL = method detection limit. The range is 4.02–9.72 ng/g lipid, which is reported for lipid-adjusted values. ^b^: Lower-chlorination PCB congeners (LPCBs). ^c^: Higher-chlorination congeners (HPCBs). ^d^: ∑PCBs means the sum of 6 indicator-PCBs (PCB28, 52, 101, 138, 153, and 180).

**Table 3:** Association of Plasma PCB burden with cognitive impairment (N = 266).

	Number of		Model 0 ^a^			Model 1 ^b^				Model 2 ^c,e^			
Variable	Normal	CoD	OR	95% CI	*p*	OR	95% CI	*p*	*p* ^d^	OR	95% CI	*p*	*p* ^d^
PCB28													
Not detected	186	43	1.00	-	ref	1.00	-		ref	1.00	-		ref
Detected	25	12	2.08	(0.94–4.39)	0.061	2.92	(1.23–6.81)	0.014	0.041	3.12	(1.19–8.11)	0.019	0.171
PCB101													
Not detected	121	35	1.00	-	ref	1.00	-		ref	1.00	-		ref
Detected	90	20	0.77	(0.41–1.41)	0.400	0.95	(0.48–1.87)	0.884	1.000	0.98	(0.46–2.04)	0.951	1.000
PCB138													
Not detected	189	52	1.00	-	ref	1.00	-		ref	1.00	-		ref
Detected	22	3	0.50	(0.11–1.50)	0.269	0.46	(0.1–1.56)	0.261	0.782	0.53	(0.10–2.00)	0.391	1.000
PCB153													
Not detected	191	53	1.00	-	ref	1.00	-		ref	1.00	-		ref
Detected	20	2	0.36	(0.06–1.29)	0.178	0.43	(0.06–1.72)	0.294	0.883	0.41	(0.06–1.84)	0.301	1.000
LPCBs ^f^													
Not detected	101	25	1.00	-	ref	1.00	-		ref	1.00	-		ref
Detected	110	30	1.10	(0.61–2.01)	0.750	1.52	(0.78–3.04)	0.224	0.672	1.55	(0.75–3.3)	0.241	1.000
HPCBs ^f^													
Not detected	175	52	1.00	-	ref	1.00	-		ref	1.00	-		ref
Detected	36	3	0.28	(0.07–0.82)	0.041	0.31	(0.07–0.99)	0.077	0.230	0.33	(0.07–1.16)	0.117	1.000
∑PCBs ^f^													
Not detected	101	25	1.00	-	ref	1.00	-		ref	1.00	-		ref
Detected	110	30	1.10	(0.61–2.01)	0.750	1.52	(0.78–3.04)	0.224	0.672	1.55	(0.75–3.3)	0.241	1.000

^a^: Model 0 was univariate logistic regression analysis. ^b^: Model 1 was adjusted by age, sex. ^c^: Model 2 was adjusted by covariates in model 1 and education level (formal education vs. without formal education), monthly income (≤1 k, 1.01–3 k, >3 k), marriage (living with a spouse vs. living without a spouse), offspring (yes vs. no), sleep quality (poor vs. general vs. well), sleep duration, and headache (yes vs. no). ^d^: n = 264, two participants were missing in ‘Sleep duration’. ^e^: *p* value was reported as Bonferroni-adjusted *p* value. ^f^: LPCBs means lower-chlorination PCB congeners (PCB 28, 52, 101). HPCBs means higher-chlorination congeners (PCB 138, 153, 180). ∑PCBs means the 6 indicator PCBs (LPCBs: PCB 28, 52, and 101; HPCBs: PCB 138, 153, and 180).

**Table 4:** Concentrations of PCBs in non-occupationally exposed populations from blood samples (ng/g Lipid) collected from other regions or countries.

Country/Regions	Year	Sample	Median (Mean)	Reference
China				
Weitang	2015–2016	Plasma	12.7 ^a^	this study
Weifang	2012	Serum	(7.1) ^b^	[33]
Weifang	2014	Serum	11 ^c^	[34]
Yitong	2014	Serum	15 ^c^	[34]
Lingshui	2014	Serum	14 ^c^	[34]
Huaihua	2014	Serum	10 ^c^	[34]
Ganzi	2014	Serum	5.9 ^c^	[34]
Canada	2011–2013	Serum	39.8 ^d^	[35]
US	2005–2007	Serum	444.9 ^a^	[36]
US	1999–2002	Serum	235 ^c^	[37]
UK	2003	Serum	103.0 ^a^	[38]
Lebanon	2018	Serum	18.9 ^a^	[39]
Iran	2016–2017	Serum	344.6 ^a^	[40]
Japan	2012	Blood	21.0 ^d^	[41]

^a^: Sum of PCBs 28, 52 101,138, 153, 180. ^b^: Sum of PCB 81, 77, 123, 118, 114, 105, 126, 167, 156, 157, 169, 170, 180 and 189. ^c^: Sum of PCBs 77, 81, 101, 105, 114, 118, 123, 126, 156, 157, 167, 169, 170, 180, and 189. ^d^: PCB153.

## Data Availability

The data that support the findings of this study are available from the corresponding author on reasonable request. Participant data without names and identifiers will be made available after approval from the corresponding authors, Soochow University and Chinese government. After publication of study findings, the data will be available for others to request. The research team will provide an email address for communication once the data are approved to be shared with others. The proposal with detailed description of study objectives and statistical analysis plan will be needed for evaluation of the reasonability to request for our data. The corresponding author, Soochow University and Chinese government will make a decision based on these materials. Additional materials may also be required during the process.

## Supplementary Materials

The following supporting information can be downloaded at: https://www.mdpi.com/article/10.3390/ijerph19126958/s1. Figure S1: The chromatogram of a real sample. Figure S2: The chromatogram of a blank sample. Table S1: Final SEM path coefficients showed the association among 4 PCBs (PCB 28, PCB 101, PCB 138, PCB 153), cognitive dysfunction and covariates for female participants.

## Abbreviations

CoD	Cognitive dysfunction
PCBs	Polychlorinated Biphenyls
dl-PCBs	dioxin-like polychlorinated biphenyls
ndl-PCBs	non-dioxin-like polychlorinated biphenyls
LPCBs	lower chlorinated polychlorinated biphenyls
HPCBs	higher chlorinated polychlorinated biphenyls
SEM	Structural Equation Modeling
AMT	Abbreviated Mental Test
NEQ	Neurotoxic Equivalent
