# Supplementary material for: Path Analysis Reveals the Direct Effect of PCB28 Exposure on Cognitive Dysfunction in Older Chinese Females
Source: Int J Environ Res Public Health. 2022 Jun 7;19(12):6958. doi: 10.3390/ijerph19126958 (PMC9222330; doi:10.3390/ijerph19126958)
Supplement: Supplementary file 1 [file ijerph-19-06958-s001.zip › ijerph-1537367-supplementary.pdf]

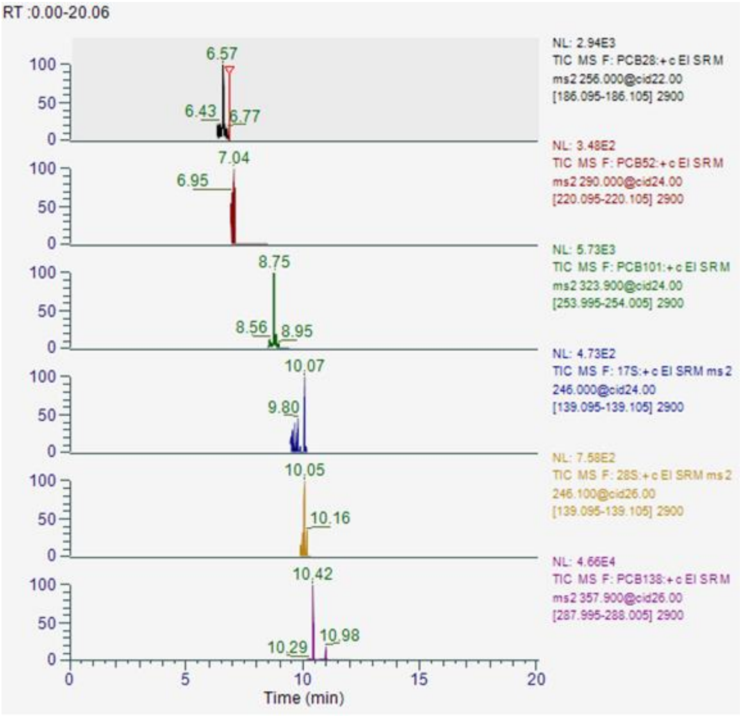
RT :0.00-20.06
NL: 2.94E3
TIC MS F: PCB28:+ c EI SRM ms2 256.000@cid22.00 [186.095-186.105] 2900
NL: 3.48E2
TIC MS F: PCB52:+ c EI SRM ms2 290.000@cid24.00 [220.095-220.105] 2900
NL: 5.73E3
TIC MS F: PCB101:+ c EI SRM ms2 323.900@cid24.00 [253.995-254.005] 2900
NL: 4.73E2
TIC MS F: 17S:+ c EI SRM ms2 246.000@cid24.00 [139.095-139.105] 2900
NL: 7.58E2
TIC MS F: 28S:+ c EI SRM ms2 246.100@cid26.00 [139.095-139.105] 2900
NL: 4.66E4
TIC MS F: PCB138:+ c EI SRM ms2 357.900@cid26.00 [287.995-288.005] 2900
Time (min)

(**a**)

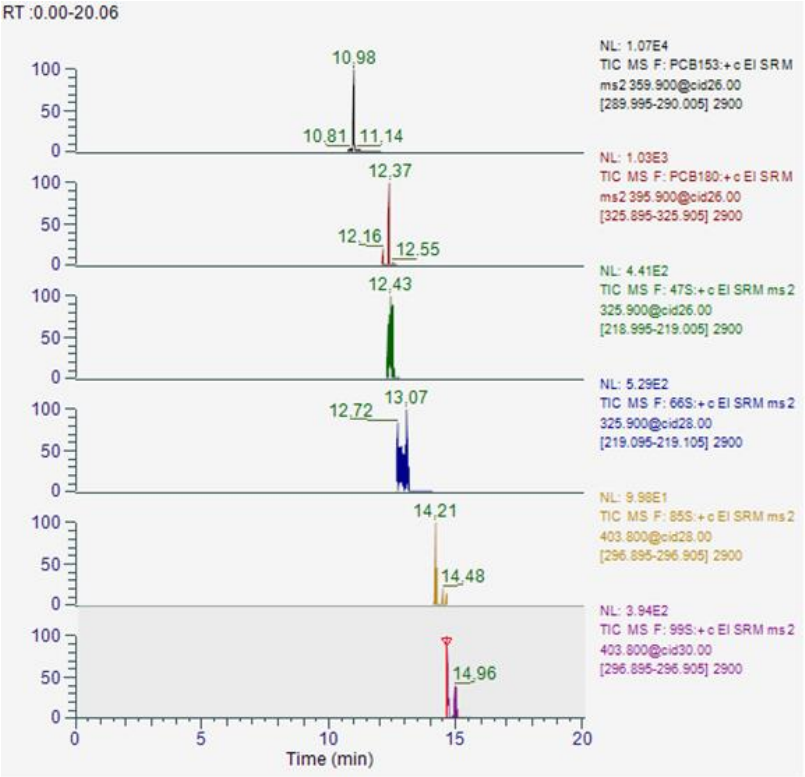
RT :0.00-20.06
NL: 1.07E4
TIC MS F: PCB153:+ c EI SRM ms2 359.900@cid26.00 [289.995-290.005] 2900
NL: 1.03E3
TIC MS F: PCB180:+ c EI SRM ms2 395.900@cid26.00 [325.895-325.905] 2900
NL: 4.41E2
TIC MS F: 47S:+ c EI SRM ms2 325.900@cid26.00 [218.995-219.005] 2900
NL: 5.29E2
TIC MS F: 66S:+ c EI SRM ms2 325.900@cid28.00 [219.095-219.105] 2900
NL: 9.98E1
TIC MS F: 85S:+ c EI SRM ms2 403.800@cid28.00 [296.895-296.905] 2900
NL: 3.94E2
TIC MS F: 99S:+ c EI SRM ms2 403.800@cid30.00 [296.895-296.905] 2900
Time (min)

(**b**)

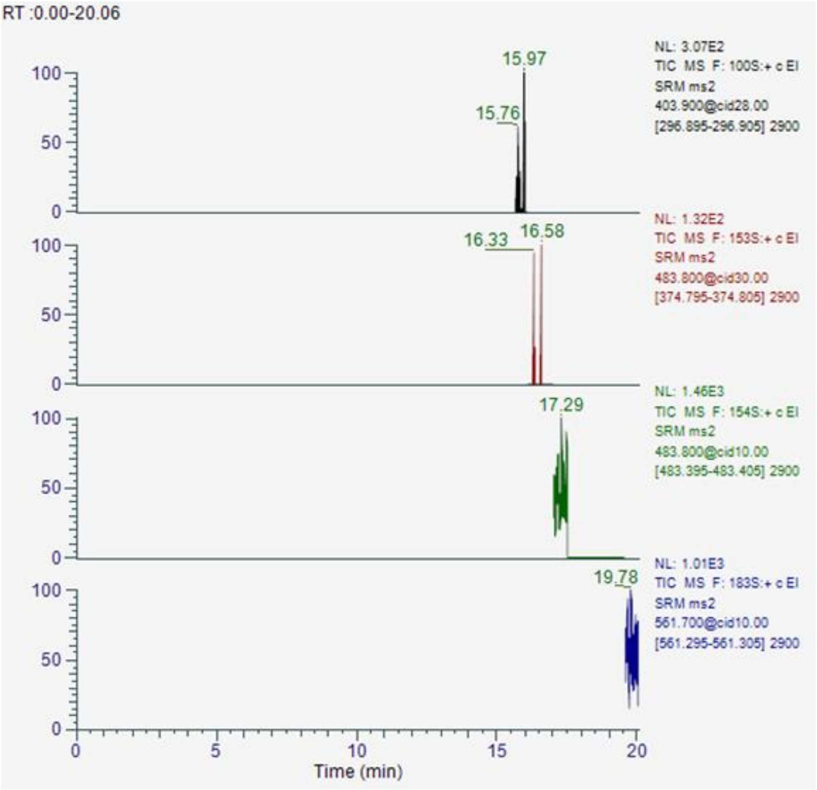

(**c**)

**Figure S1.** The chromatogram of a real sample.

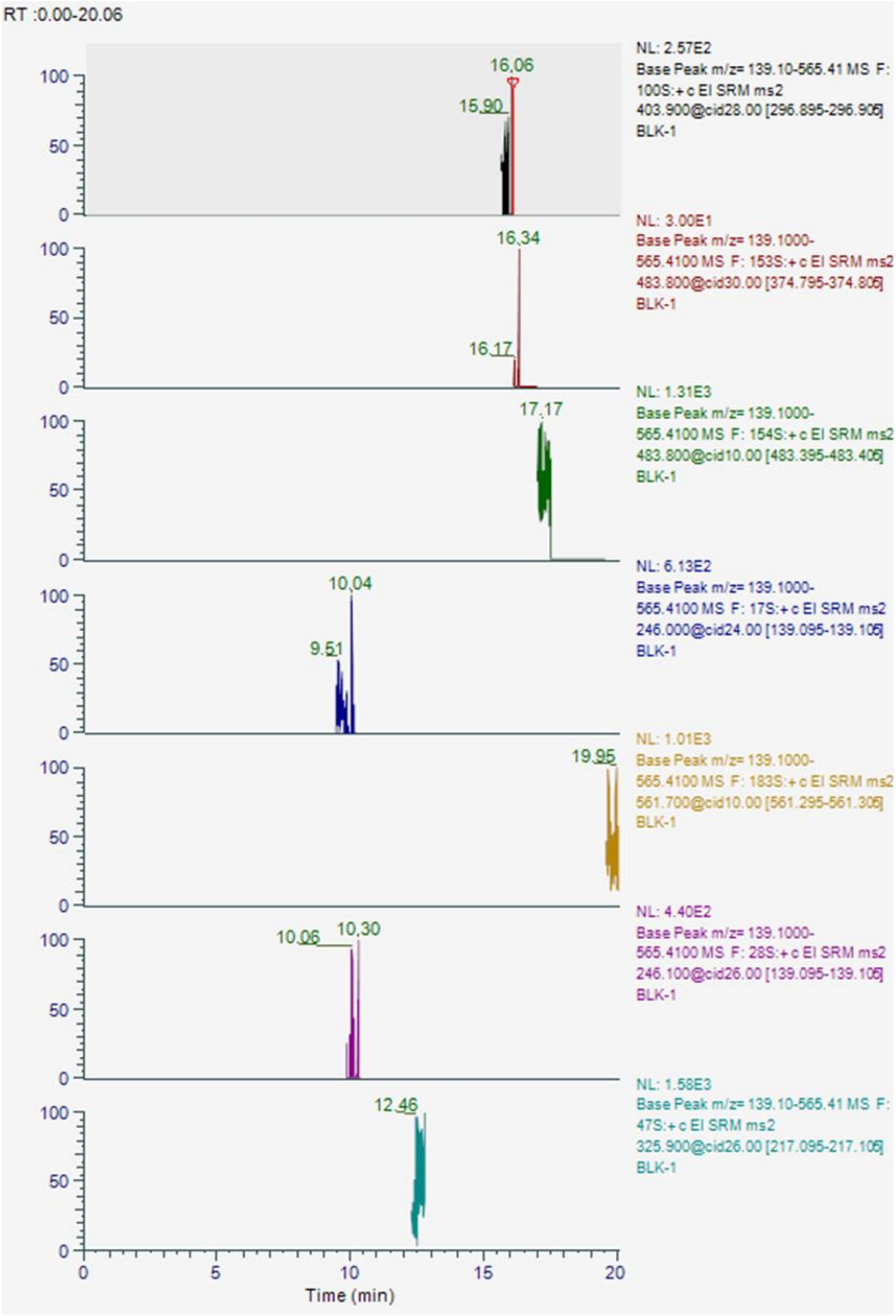
RT :0.00-20.06
NL: 2.57E2
Base Peak m/z= 139.10-565.41 MS F: 100S:+ c EI SRM ms2 403.900@cid28.00 [296.895-296.905]
BLK-1
16.06
15.90
NL: 3.00E1
Base Peak m/z= 139.1000-565.4100 MS F: 153S:+ c EI SRM ms2 483.800@cid30.00 [374.795-374.805]
BLK-1
16.34
16.17
NL: 1.31E3
Base Peak m/z= 139.1000-565.4100 MS F: 154S:+ c EI SRM ms2 483.800@cid10.00 [483.395-483.405]
BLK-1
17.17
NL: 6.13E2
Base Peak m/z= 139.1000-565.4100 MS F: 17S:+ c EI SRM ms2 246.000@cid24.00 [139.095-139.105]
BLK-1
10.04
9.51
NL: 1.01E3
Base Peak m/z= 139.1000-565.4100 MS F: 183S:+ c EI SRM ms2 561.700@cid10.00 [561.295-561.305]
BLK-1
19.95
NL: 4.40E2
Base Peak m/z= 139.1000-565.4100 MS F: 28S:+ c EI SRM ms2 246.100@cid26.00 [139.095-139.105]
BLK-1
10.06
10.30
NL: 1.58E3
Base Peak m/z= 139.10-565.41 MS F: 47S:+ c EI SRM ms2 325.900@cid26.00 [217.095-217.105]
BLK-1
12.46
Time (min)

(a)

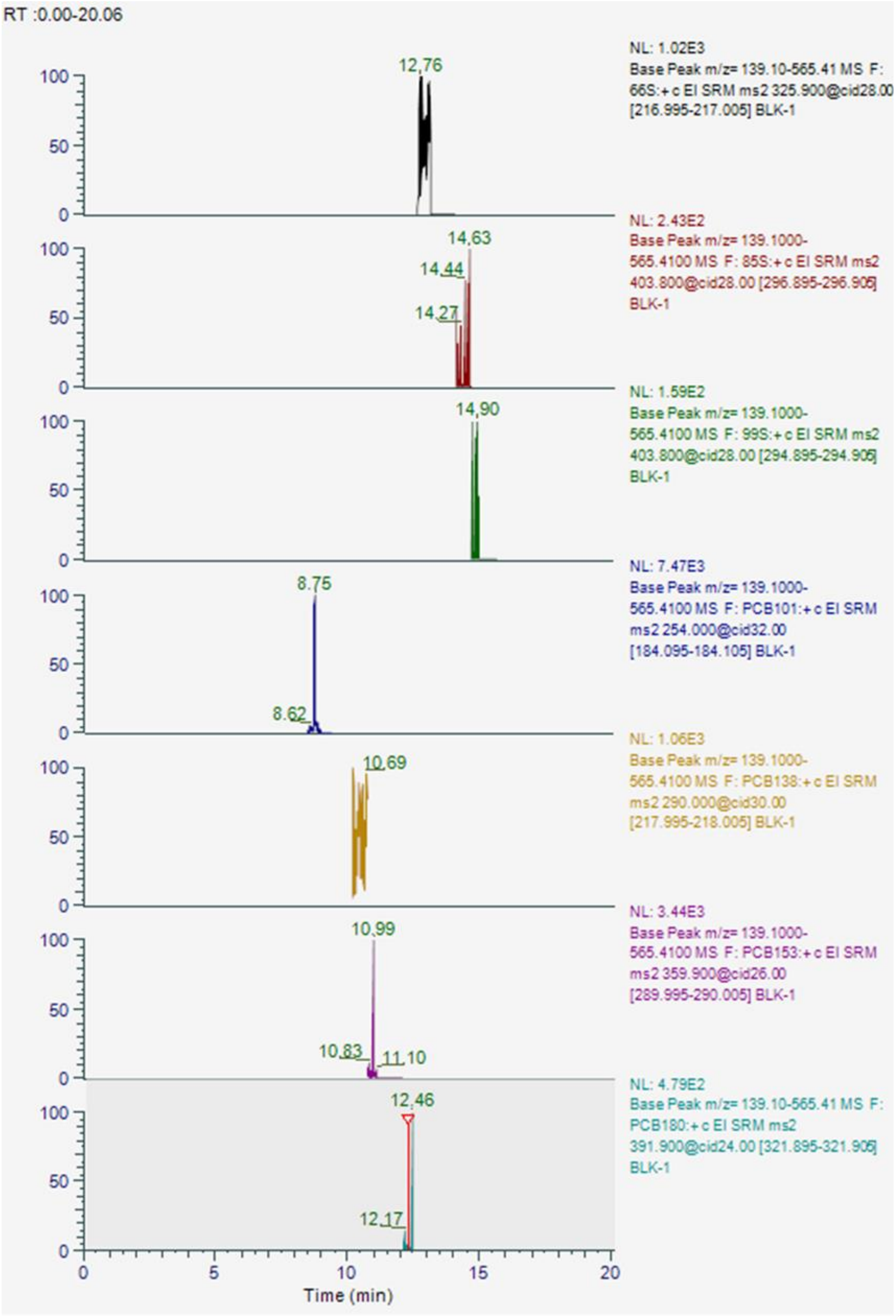
RT :0.00-20.06
NL: 1.02E3
Base Peak m/z= 139.10-565.41 MS F: 66S:+ c EI SRM ms2 325.900@cid28.00 [216.995-217.005] BLK-1
12,76
NL: 2.43E2
Base Peak m/z= 139.1000-565.4100 MS F: 85S:+ c EI SRM ms2 403.800@cid28.00 [296.895-296.905] BLK-1
14,63
14,44
14,27
NL: 1.59E2
Base Peak m/z= 139.1000-565.4100 MS F: 99S:+ c EI SRM ms2 403.800@cid28.00 [294.895-294.905] BLK-1
14,90
NL: 7.47E3
Base Peak m/z= 139.1000-565.4100 MS F: PCB101:+ c EI SRM ms2 254.000@cid32.00 [184.095-184.105] BLK-1
8.75
8.62
NL: 1.06E3
Base Peak m/z= 139.1000-565.4100 MS F: PCB138:+ c EI SRM ms2 290.000@cid30.00 [217.995-218.005] BLK-1
10,69
NL: 3.44E3
Base Peak m/z= 139.1000-565.4100 MS F: PCB153:+ c EI SRM ms2 359.900@cid26.00 [289.995-290.005] BLK-1
10,99
10,83
11,10
NL: 4.79E2
Base Peak m/z= 139.10-565.41 MS F: PCB180:+ c EI SRM ms2 391.900@cid24.00 [321.895-321.905] BLK-1
12,46
12,17
0
50
100
0
5
10
15
20
Time (min)

(**b**)

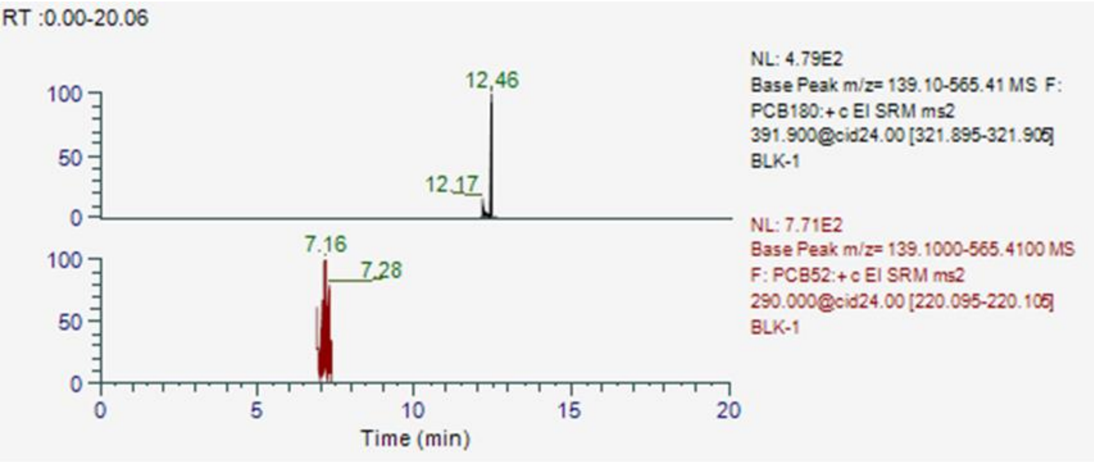


(c)

**Figure S2. The chromatogram of a blank sample.**

**Table S1. Final SEM path coefficients showed the association among 4 PCBs (PCB 28, PCB 101, PCB 138, PCB 153), cognitive dysfunction and covariates for female participants.**

| Path | Path coefficient | | *p* |
|---|---|---|---|
| | Estimate | Standard Error (SE) | |
| **Females & Age ≤ 80 (N=125)** [a] | | | |
| Age → CoD | 0.059 | 0.029 | 0.044 |
| Education level → CoD | -0.591 | 0.333 | 0.076 |
| Monthly income → CoD | -0.052 | 0.303 | 0.864 |
| Marriage → CoD | -0.197 | 0.447 | 0.659 |
| Children → CoD | -0.265 | 0.282 | 0.347 |
| Sleep duration → CoD | 0.152 | 0.153 | 0.321 |
| Sleep quality → CoD | -0.348 | 0.134 | 0.010* |
| Headache → CoD | 0.321 | 0.128 | 0.012* |
| PCB 28 → CoD | 0.670 | 0.339 | 0.048* |
| PCB 101 → CoD | 0.168 | 0.295 | 0.570 |
| PCB 138 → CoD | -0.423 | 1.103 | 0.701 |
| PCB 153 → CoD | -0.236 | 1.114 | 0.832 |
| PCB 28 → Headache | -0.509 | 0.415 | 0.220 |
| PCB 101 → Headache | -0.177 | 0.307 | 0.565 |
| PCB 138 → Headache | -0.342 | 0.476 | 0.473 |
| PCB 153 → Headache | 0.140 | 0.540 | 0.795 |

| | | | |
|---|---|---|---|
| Age → Sleep quality | 0.032 | 0.027 | 0.236 |

a: model fit index ($X^2$ *p* value = 0.463, CFI = 1.000, RMSEA = 0.000).

Criterion for good fit: $X^2$ *p* value > 0.05, CFI > 0.95, RMSEA < 0.05.

* $p < 0.05$
